# Evaluation of the Effect of Blood Transfusion on Retinopathy of Prematurity at a Tertiary Care Center in Western Saudi Arabia

**DOI:** 10.7759/cureus.24495

**Published:** 2022-04-26

**Authors:** Lina H Raffa, Wasayf Aljohani

**Affiliations:** 1 Ophthalmology, King Abdulaziz University Hospital, Jeddah, SAU; 2 Faculty of Medicine, King Abdulaziz University Hospital, Jeddah, SAU

**Keywords:** low birth weight, rbc, mortality, infants, prematurity, blood transfusion, retinopathy of prematurity

## Abstract

Introduction

We aim to study if there is an association between blood transfusions and the development and severity of retinopathy of prematurity (ROP). We also aim to explore the association with other clinical outcomes.

Methods

A cohort of 291 infants admitted to our neonatal intensive care unit (NICU) was retrospectively analyzed. The number and volume of RBC transfusions on Day 7 and Day 30 were recorded. Clinical outcomes including ROP, necrotizing enterocolitis (NEC), bronchopulmonary dysplasia (BPD), and sepsis were noted.

Results

The mean gestational age (GA) and birth weight (BW) of the evaluated infants were 28 ± 2.2 weeks and 1062 ± 242 g, respectively. One hundred and eighteen infants were transfused at a median of nine days post GA. Compared to non-transfused infants, those who were transfused had a lower GA, a lower BW, a longer stay in the NICU, and received significantly more artificial ventilation. These infants also had a higher number of comorbidities, including sepsis and intraventricular hemorrhage. The number and volume of RBCs at Day 30 were significantly higher in infants with any stage of ROP than in those without ROP.

Conclusion

A higher frequency and volume of RBC transfusion were associated with an increased risk of ROP development. Whether this is a direct consequence of blood transfusion or the infants being at risk due to prematurity or low BW remains to be determined.

## Introduction

Retinopathy of prematurity (ROP) is a potentially blinding eye disease that primarily affects prematurely born infants, mainly neonates who are born before <=30 weeks and who typically weigh <=1500 g [1]. In 2008, the estimated incidence of ROP in 147 infants in Saudi Arabia was 56% [2]. Moreover, a multicenter trial on 4099 preterm infants revealed that 65.8% of the infants developed ROP, and 81.6% of those who weighed less than 1000 g [3]. Regarding the etiology of ROP, the major risk factors for the disorder are low birth weight (BW), low gestational age (GA), and oxygen therapy [4-6]. In addition, several studies have suggested that blood transfusions, mechanical ventilation, and sepsis are the other factors that contribute to an increased incidence of ROP in premature infants [5,7].

In the neonatal intensive care unit (NICU), premature infants are very likely to develop anemia and require the transfusion of at least one unit of RBCs [8]. The smaller the infants, the more likely the requirement for frequent transfusion. Up to 94% of very low birth weight infants (VLBW; BW: <1500 g) and as high as 95% of extremely low birth weight infants (ELBW; BW: <1000 g) require at least one blood transfusion during their hospital stay [9].

Transfusion of RBCs remains a topic of study to determine its likely contribution to a few of the major causes of mortality and morbidity in premature infants, including bronchopulmonary dysplasia (BPD), intraventricular hemorrhage (IVH), and necrotizing enterocolitis (NEC) [10-12]. In particular, associations between RBC transfusion and the development of ROP have been described [13-15]. In recent times, several retrospective studies have shown that the number and frequency of transfusions correlate with the risk of developing threshold ROP [15-17]. Moreover, it has been reported that the relationship between blood transfusion and ROP is secondary to a sudden increase in oxygen delivery to the retina, downregulation of vascular endothelial growth factor, and oxidative damage to the retinal cells owing to iron overload [17-19]. 

There is a lack of guidelines on ideal transfusion conditions for premature infants, despite packed RBCs being the most commonly transfused blood components in these infants, particularly VLBW premature infants who frequently require multiple blood transfusions in the NICU [8]. Neonatal transfusion guidelines greatly vary globally and among institutions; nevertheless, attempts are being made to establish evidence-based guidelines for this purpose [20]. This retrospective study aimed to analyze a cohort of premature infants in Saudi Arabia and investigate the relationship between blood transfusion and severity of ROP and the association of RBC transfusion with other clinical outcomes at our institute.

## Materials and methods

Study setting and subjects

This retrospective cohort study involved a review of electronic medical records of infants in the NICU at King Abdulaziz University Hospital (KAUH), Jeddah, Saudi Arabia. All infants admitted to the NICU with a BW of <1500g or born ≤30 weeks between 2013 and 2019 were enrolled. The exclusion criteria were as follows: the presence of chromosomal anomalies that might impair normal physiological function, incomplete ROP data, and death within seven days of birth.

Descriptive and clinical variables were recorded for all infants. Furthermore, the numbers and total volume of RBC transfusions within 7 and 30 days of birth and the date of the first transfusion were recorded for all infants. In addition, the following perinatal variables were collected: sex, GA, BW, initial hemoglobin level, and 1- and 5-minute Apgar scores. The primary outcomes were in-hospital mortality during the first seven days and morbidities of NEC, ROP, sepsis, and BPD. Trained ophthalmologists screened all eligible infants for ROP by using the ROP screening criteria of the American Academy of Pediatrics [21]. ROP was classified according to the international classification of ROP [22]. All treatments were administered according to the Early Treatment for Retinopathy of Prematurity study guidelines [23]. VLBW was defined as BW of 1001-1499 g, and ELBW as BW of <1000 g.

A widely adopted transfusion protocol for premature infants is used in our NICU [14]. The indications for RBC transfusion followed in our institution are as follows: (1) shock due to blood loss; (2) hematocrit of <20% and a reticulocyte count of <100,000/µL in asymptomatic patients; (3) hematocrit of <30% on oxygen dependency, ventilator dependency, marked apnea or bradycardia, poor weight gain despite the provision of adequate nutrition, and persistent tachycardia for more than 24 hours in symptomatic infants; (4) symptomatic infants undergoing surgery; and (5) hematocrit of <33% while receiving >35% supplemental oxygen or requirement of ventilator assistance for breathing. A volume of 15 mL/kg of BW of packed RBCs was transfused over three hours.

Statistical methodology

IBM SPSS version 23 (IBM Corp., Armonk, NY, USA) was used for statistical analysis. Simple descriptive statistics were used to define study variable characteristics in the form of counts and percentages for categorical and nominal variables and mean and SD values for continuous variables. Infants were grouped based on whether they developed ROP. Those who developed ROP were further categorized per the severity of the final stage reached. To evaluate the relationship between categorical variables, the Chi-squared test was used. Independent t-tests and one-way analysis of variance, with the least significant difference (LSD) as a posthoc test, were used to compare two groups and more than two groups. These tests were done with the assumption of normal distribution. Otherwise, Welch’s t-test and the Games-Howell posthoc test were used as alternatives to the LSD test for two and multiple groups, respectively. Since the dispersion of the blood transfusion volume was high, a nonparametric test (median test) was used to determine if there was a difference in median levels of blood transfusion relative to the presence of ROP. Dependent study variables were defined as a binary outcome. A binary logistic regression model with backward conditional elimination with enter criteria = 0.05 and elimination = 0.10 was used to determine the significant predictors of any dependent study variable with 95% CIs. A conventional p-value of <0.05 was the criteria to reject the null hypothesis.

Consent

The study was conducted per the Declaration of Helsinki and its tenets and was approved by the Research Ethics Committee at KAUH (approval number: 162-22).

## Results

The data for 305 infants who were admitted to the NICU at KAUH between 2013 and 2019 were reviewed in this study. Premature infants with a GA of <31 weeks and or BW of <1500 g with ROP outcomes were included in the study (n = 291). Fourteen infants that did not meet the inclusion criteria (n = 12) or showed chromosomal abnormalities (n = 2) were excluded. Among the 291 infants included in this study, 162 and 119 were VLBW and ELBW infants, respectively, while 136 infants were males (46.7%). The mean GA and BW were 28 ± 2.2 weeks and 1062 ± 242 g, respectively. A total of 147 infants were of Saudi nationality (50.9%). The average initial hemoglobin level was 10.13 ± 2.7 g/dl. Blood transfusion was conducted at a median of nine days of post GA. The mean number of RBC transfusions and volume of RBCs transfused were five times and 98 mL/infant, respectively. Table 1 shows the characteristics of all the included infants.

**Table 1:** Characteristics of the 291 infants along with the number and volume of RBC transfusions within 7 and 30 days of life. GA: Gestational age; BW: Birth weight; Hgb: Hemoglobin.

Variables	Mean	SD	Minimum	Maximum
GA (weeks)	28.65	2.2	23	39
BW (g)	1061.67	242.2	527.0	1840.0
Initial Hgb (g/dL)	10.13	2.7	6.30	19.50
Apgar score 1 min	5.32	2.2	0	10
Apgar score 5 min	7.72	1.6	1	10
Duration of hospitalization (days)	67.57	61.5	7.0	545.0
No. of transfusions Day 7	1.26	1.5	0	7
No. of transfusions Day 30	3.06	2.6	0	11
Transfusion volume (mL) Day 7	25.75	41.8	0	320
Transfusion volume (mL) Day 30	54.82	53.1	0	320

The transfused babies showed a lower GA, lower BW, longer NICU stay, and greater usage of artificial ventilation than non-transfused infants. These patients also showed higher comorbidities, including sepsis and IVH (Table 2).

**Table 2:** Comparison of transfused versus non-transfused premature infants. NICU: Neonatal intensive care unit; VLBW: Very low birth weight; ELBW: Extremely low birth weight; NBW: Normal birth weight; IVH: Intraventricular hemorrhage; ROP: Retinopathy of prematurity; NEC: Necrotizing enterocolitis; BPD: Bronchopulmonary dysplasia. a: significant using independent t-test at <0.05 level. b: significant using Chi-squared test at <0.05 level.

Variables		Total	Blood transfusion		p-value
			Yes	No	
Birth weight		291	993.79 ± 232.4	1112.78 ± 237.5	<0.001^a^
Gestational age (weeks)		291	28.21 ± 2.2	28.98 ± 2.1	0.003^a^
Length of stay in NICU		291	76.51 ± 62.9	60.84 ± 59.7	0.031^a^
Birth weight	VLBW	162	56 (34.6%)	106 (65.4%)	0.004^b^
	ELBW	119	65 (54.6%)	54 (45.4%)	
	NBW	10	4 (40.0%)	6 (60.0%)	
Sex	Male	136	57 (41.9%)	79 (58.1%)	0.736
	Female	155	68 (43.9%)	87 (56.1%)	
IVH	Yes	80	44 (55.0%)	36 (45.0%)	0.011^b^
	No	211	81 (38.4%)	130 (61.6%)	
Sepsis	Yes	99	61 (61.6%)	38 (38.4%)	<0.001^b^
	No	192	64 (33.3%)	128 (66.7%)	
Artificial ventilation	Yes	280	125 (44.6%)	155 (55.4%)	0.003^b^
	No	11	0 (0.0%)	11 (100.0%)	
Any stage ROP	Yes	90	43 (47.8%)	47 (52.2%)	0.261
	No	199	81 (40.7%)	118 (59.3%)	
Treatable ROP	Yes	20	10 (50.0%)	10 (50.0%)	0.910
	No	70	34 (48.6%)	36 (51.4%)	
Deceased	Yes	16	11 (68.8%)	5 (31.3%)	0.032^b^
	No	275	114 (41.5%)	161 (58.5%)	
NEC	Yes	28	14 (50.0%)	14 (50.0%)	0.428
	No	263	111 (42.2%)	152 (57.8%)	
BPD	Yes	14	6 (42.9%)	8 (57.1%)	0.994
	No	277	119 (43.0%)	158 (57.0%)	

Binary logistic regression analysis revealed that low BW and the presence of sepsis were the most significant variables associated with blood transfusion for infants (p = 0.001). In addition, the number of RBCs on days 7 and 30, and the transfusion volume on Day 30 were significantly higher in infants with ROP of any stage than in infants without ROP (Table 3).

**Table 3:** Association of the number and volume of blood transfusions by Day 7 and Day 30 with outcome variables. D7: Day 7; D30: Day 30; tVol: Total volume; IVH: Intraventricular hemorrhage; ROP: Retinopathy of prematurity; NEC: Necrotizing enterocolitis; BPD: Bronchopulmonary dysplasia; VLBW: Very low birth weight; ELBW: Extremely low birth weight. a: significant using independent t-test at <0.05 level. b: significant using Welch's t-test at <0.05 level.

Variables		Total	Number of transfusions by D7	Number of transfusions by D30	tVol. of RBCs by D7	tVol. Of RBCs by D30
Any stage ROP	Yes	38	1.74 ± 1.7	4.24 ± 2.9	37.64 ± 55.5	77.12 ± 68.2
	No	72	1.00 ± 1.3	2.43 ± 2.2	19.40 ± 31.4	42.89 ± 39.1
p-value			0.012^a^	0.002^b^	0.067	0.006^b^
Treatable ROP	Yes	9	2.33 ± 1.9	5.78 ± 3.5	40.50 ± 41.0	82.50 ± 61.8
	No	30	1.50 ± 1.6	3.80 ± 2.6	35.53 ± 59.2	75.13 ± 69.8
p-value			0.194	0.073	0.816	0.778
NEC	Yes	12	1.67 ± 1.6	4.67 ± 3.2	52.92 ± 61.1	104.33 ± 62.7
	No	99	1.21 ± 1.5	2.87 ± 2.5	22.46 ± 38.0	48.81 ± 48.8
p-value			0.314	0.023^b^	0.117	<0.001^a^
IVH	Yes	38	1.71 ± 1.7	4.42 ± 2.9	28.75 ± 33.3	67.51 ± 49.7
	No	73	1.03 ± 1.3	2.36 ± 2.1	24.19 ± 45.8	48.21 ± 53.9
p-value			0.034^b^	<0.001^b^	0.588	0.069
BPD	Yes	5	1.60 ± 1.1	3.60 ± 2.4	23.60 ± 19.2	52.60 ± 15.9
	No	106	1.25 ± 1.5	3.04 ± 2.6	25.85 ± 42.6	54.92 ± 54.2
p-value			0.6	0.639	0.907	0.924
Sepsis	Yes	51	1.57 ± 1.5	3.80 ± 2.7	31.93 ± 48.7	67.64 ± 59.5
	No	60	1.00 ± 1.4	2.43 ± 2.3	20.50 ± 34.4	43.92 ± 44.6
p-value			0.042^a^	0.006^b^	0.152	0.018^a^
Birth weight	VLBW	49	0.71 ± 0.9	2.12 ± 1.8	25.55 ± 54.3	49.55 ± 57.7
	ELBW	59	1.69 ± 1.7	3.92 ± 2.9	25.13 ± 29.2	59.87 ± 50.2
p-value			<0.001^b^	<0.001^b^	0.959	0.322

Although higher frequency and volume were also noted in infants with treatable ROP, this difference was not statistically significant.

**Figure 1:**
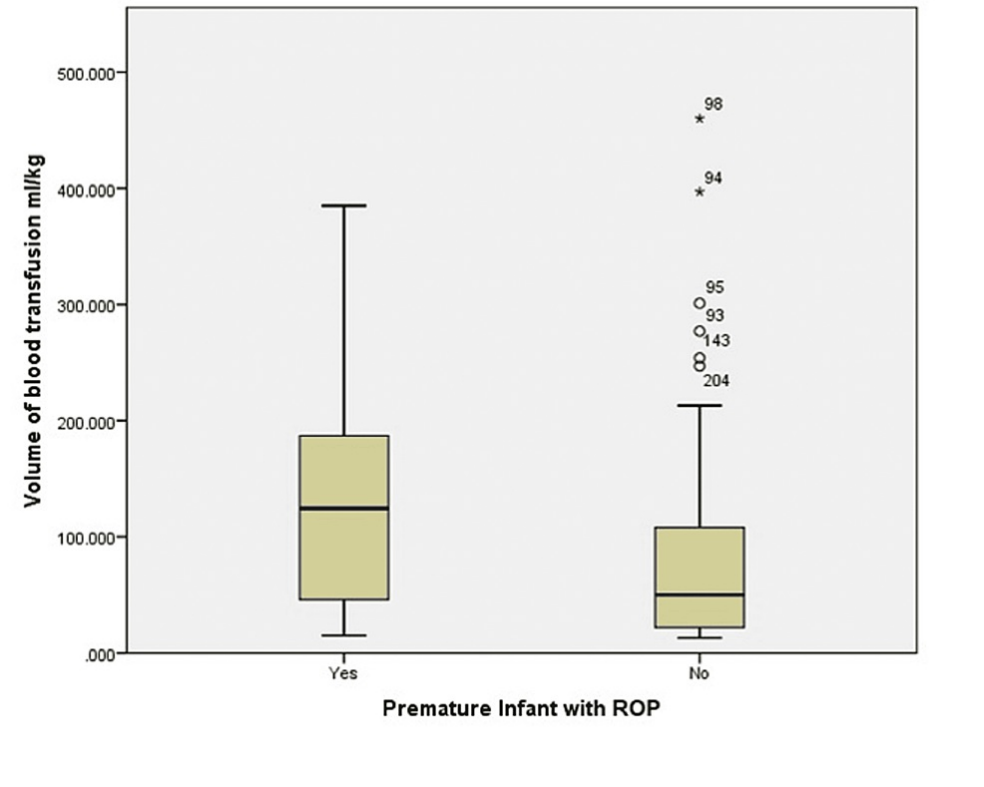
Box plot representing the relationship between the volume of blood transfusion and retinopathy of prematurity.

Although higher frequency and volume were also noted in infants with treatable ROP, this difference was not statistically significant. A total of 90 infants showed ROP, while 20 infants required treatment. The worst ROP was diagnosed as stages 1, 2, and 3 in 70.8%, 19.1%, and 10.1% of the infants, respectively, mainly in zones 2 and 3 (47.7% and 51.1%). The median blood transfusion volume was 59 mL/kg (13-460 mL). Premature infants with ROP of any stage were more likely to have received higher volumes of blood transfusion than infants with no ROP (p = 0.016) (Figure 1).

Infants with the worst stage ≥ 2 in either eye were more likely to show a lower BW (p = 0.001), lower GA (p = 0.007), longer NICU duration (p = 0.019), and higher incidence of BPD (p = 0.03) than those with milder ROP (<stage 2) (Table 4).

**Table 4:** Comparison of outcome variables between infants with mild ROP (under stage 2) and those with severe ROP (over stage 2). NICU: Neonatal intensive care unit; VLBW: Very low birth weight; ELBW: Extremely low birth weight; NBW: Normal birth weight; IVH: Intraventricular hemorrhage; ROP: Retinopathy of prematurity; NEC: Necrotizing enterocolitis; BPD: Bronchopulmonary dysplasia. a: significant using independent t-test at <0.05 level. b: significant using Welch's t-test at <0.05 level. c: significant using Chi-squared test at <0.05 level.

Variables		Total	Worst ROP stage in either eye		p-value
			Stage 1	Stage ≥2	
Birth weight		89	999.97 ± 253.6	802.65 ± 146.7	<0.001^b^
Gestational age (weeks)		89	27.73 ± 1.8	26.62 ± 1.6	0.007^a^
Length of stay in NICU		89	74.25 ± 41.1	124.00 ± 98.7	0.019^b^
Weight gain at 4 weeks		75	223.45 ± 218.0	165.00 ± 131.5	0.275
Number of blood transfusion		39	6.70 ± 4.7	7.33 ± 4.1	0.720
Sex	Male	38	24 (63.2%)	14 (36.8%)	0.172
	Female	51	39 (76.5%)	12 (23.5%)	
Birth weight	VLBW	31	28 (90.3%)	3 (9.7%)	0.006^c^
	ELBW	56	33 (58.9%)	23 (41.1%)	
	NBW	2	2 (100.0%)	0 (0.0%)	
IVH	Yes	31	20 (64.5%)	11 (35.5%)	0.342
	No	58	43 (74.1%)	15 (25.9%)	
Sepsis	Yes	34	24 (70.6%)	10 (29.4%)	0.974
	No	55	39 (70.9%)	16 (29.1%)	
Artificial ventilation	Yes	87	62 (71.3%)	25 (28.7%)	0.513
	No	2	1 (50.0%)	1 (50.0%)	
NEC	Yes	10	6 (60.0%)	4 (40.0%)	0.426
	No	79	57 (72.2%)	22 (27.8%)	
BPD	Yes	8	3 (37.5%)	5 (62.5%)	0.030^c^
	No	81	60 (74.1%)	21 (25.9%)	
Deceased	Yes	5	5 (100.0%)	0 (0.0%)	0.139
	No	84	58 (69.0%)	26 (31.0%)	

## Discussion

Transfusion of RBCs is a commonly employed life-saving therapeutic modality for anemic neonates and infants, particularly prematurely born infants. While blood transfusions have been previously implicated in the etiology of ROP, association does not prove causation; nevertheless, the role of frequent transfusions in ROP [24,25] may be explained by some factors. In our study, infants who received transfusions were younger and smaller in weight, required longer periods of hospitalization, and showed a higher incidence of sepsis, IVH, and need for artificial ventilation support than non-transfused infants. In the binary logistic regression analysis, low BW and the presence of sepsis were the most significant variables associated with blood transfusion in infants.

The relation between RBC transfusion and adverse clinical outcomes in premature infants has been studied extensively. However, since premature infants are demonstrably prone to morbidities such as BPD, NEC, sepsis, and IVH, the distinction between transfusion as an independent risk factor resulting in a specific adverse outcome or a marker of disease severity is difficult to establish. Nevertheless, the disturbance of microcirculatory regulation caused by RBC transfusion has been suggested to compromise tissue oxygenation in histological studies [26]. 

Somani A et al. had previously reported the association between mortality and RBC transfusion across all age groups in critically ill patients [26]. While, dos Santos AM et al. reported that RBC transfusions in the first 28 days of life, regardless of the frequency, were associated with a 50% greater risk of in-hospital mortality in VLBW infants in comparison with the risk in infants that did not receive transfusion [28]. This finding agrees with the results of our study, in which babies that received transfusions showed significantly higher mortality rates than non-transfused babies. This association is considered to be a manifestation of transfusion-related immunomodulation, which through proinflammatory mechanisms, involves serious effects attributable to blood transfusion [29]. In a prospective comparison performed after adjusting for several confounders that included eight neonatal centers, a mortality relative risk of 1.49 was established for VLBW infants who received one transfusion, versus a mortality relative risk of 1.89 for those who received two transfusions. In their study population, infants received 3.4 transfusions per infant on average [28], while the average number of transfusions at our institute was five transfusions per infant.

Previous reports have established an association between ROP development and RBC transfusion [12,30,31]. The possible mechanisms underlying the complications associated with RBC transfusions in preterm infants include an increased incidence of oxidative injury secondary to the increase in iron levels or the release of inflammatory mediators from stored blood products [32]. Dani C et al. [33] also demonstrated that the iron derived from RBC transfusions independently contributed to ROP development, providing evidence of the key roles of free radicals and iron overload in the pathogenesis of this condition. Similar to the findings of our study, Valieva OA et al. also [34] observed that the incidence of severe ROP was higher in VLBW infants that received transfusions; however, the increase in the number of blood transfusions noted in stage 2 and higher cases was non-significant in comparison with the incidence in the milder cases (<stage 2). Nevertheless, infants with severe ROP showed significantly lower GA, lower BW, and longer NICU stays than infants with milder ROP. Fortes Filho JB et al. [35] demonstrated that higher volumes of RBC transfusion were associated with ROP, consistent with our results showing significantly higher volumes of RBCs transfused at Day 30 (77.12 ± 68.2 mL) in infants with ROP of any stage in comparison with infants showing no ROP (42.89 ± 39.1 mL; p = 0.006). Our study also demonstrated significantly higher numbers of blood transfusions in infants with ROP of any stage at both days 7 and 30 in comparison with infants with no ROP. Higher frequency and volume were also noted in infants with treatable ROP; however, that difference was not statistically significant.

BPD has been shown to be associated with RBC transfusion [34,36] for neonatal patients. Chen HL et al. [36] demonstrated that VLBW infants who received transfusions of more than 30 mL/kg were risk factors for developing chronic lung disease. However, our results were not consistent with this finding. Moreover, a retrospective case-control study also showed an increased likelihood of IVH in infants that received transfusions [37], consistent with the findings of our study. RBC transfusion in preterm infants has also been linked to an increased risk of NEC. Blau J et al. and Mally P et al. [38,39] described a group of premature infants who developed NEC within 48 hours following RBC transfusion. Increased incidence of NEC in transfused babies was not demonstrated in our study.

To our knowledge, this is the first study to analyze the associations between blood transfusion protocols and ROP in Saudi Arabia. The first limitation of this study is its retrospective observational nature, which precluded the ability to control for various confounding factors. For example, prematurity itself is a predisposing factor for the more severe gut, lung, and brain immaturity, leading to severe lung disease, longer periods of mechanical ventilation, and higher incidence rates of IVH, NEC, and sepsis. The second limitation was the lack of investigation of the storage age of blood transfused to the infants, which has been shown to be a risk factor in previous studies. The mean lifespan of RBCs transfused into preterm infants has been reported to be much shorter than that of those transfused into adults, leading to an accelerated breakdown of RBCs and iron overload in these infants [40].

## Conclusions

In conclusion, a higher frequency and volume of RBC transfusion were associated with an increased risk of ROP development. In our study, infants who received blood transfusions showed a higher incidence of adverse outcomes, including sepsis, IVH, and need for artificial ventilation support, than non-transfused infants. In addition, the regression analysis performed in our study indicated that a higher number of RBC transfusions was associated with an increased incidence of low BW and sepsis. These findings highlight the need for additional large-cohort studies to set clearer RBC transfusion practices in this vulnerable group of infants and better understand the association between blood transfusions and the development and severity of ROP. Despite the life-saving potential of RBC transfusions, precision in the preparation, use of this therapy, and application offer the greatest potential for yielding safer patient outcomes and more effective therapy.
